# Anti-N-methyl-D-aspartate receptor encephalitis with serum anti-thyroid antibodies and IgM antibodies against Epstein-Barr virus viral capsid antigen: a case report and one year follow-up

**DOI:** 10.1186/1471-2377-11-149

**Published:** 2011-11-29

**Authors:** Chun-Ling Xu, Lei Liu, Wei-Qin Zhao, Ji-Mei Li, Rui-Jin Wang, Shu-Hui Wang, De-Xin Wang, Mei-Yun Liu, Shan-Shan Qiao, Jia-Wei Wang

**Affiliations:** 1 Department of Neurology, Beijing Friendship Hospital, Capital Medical University, No.95 Yong'An Road, Beijing, 100050, China

## Abstract

**Background:**

Anti-N-methyl-D-aspartate receptor encephalitis is an increasingly common autoimmune disorder mediated by antibodies to certain subunit of the N-methyl-D-aspartate receptor. Recent literatures have described anti-thyroid and infectious serology in this encephalitis but without follow-up.

**Case presentation:**

A 17-year-old Chinese female patient presented with psychiatric symptoms, memory deficits, behavioral problems and seizures. She then progressed through unresponsiveness, dyskinesias, autonomic instability and central hypoventilation during treatment. Her conventional blood work on admission showed high titers of IgG antibodies to thyroglobulin, thyroid peroxidase and IgM antibodies to Epstein-Barr virus viral capsid antigen. An immature ovarian teratoma was found and removal of the tumor resulted in a full recovery. The final diagnosis of anti-N-methyl-D-aspartate receptor encephalitis was made by the identification of anti-N-methyl-D-aspartate receptor antibodies in her cerebral spinal fluid. Pathology studies of the teratoma revealed N-methyl-D-aspartate receptor subunit 1 positive ectopic immature nervous tissue and Epstein-Barr virus latent infection. She was discharged with symptoms free, but titers of anti-thyroid peroxidase and anti-thyroglobulin antibodies remained elevated. One year after discharge, her serum remained positive for anti-thyroid peroxidase and anti-N-methyl-D-aspartate receptor antibodies, but negative for anti-thyroglobulin antibodies and IgM against Epstein-Barr virus viral capsid antigen.

**Conclusions:**

Persistent high titers of anti-thyroid peroxidase antibodies from admission to discharge and until one year later in this patient may suggest a propensity to autoimmunity in anti- N-methyl-D-aspartate receptor encephalitis and support the idea that neuronal and thyroid autoimmunities represent a pathogenic spectrum. Enduring anti-N-methyl-D-aspartate receptor antibodies from admission to one year follow-up but seroreversion of Epstein-Barr virus viral capsid antigen IgM may raise the important issue of elucidating the triggers and boosters of anti- N-methyl-D-aspartate receptor encephalitis.

## Background

Anti-N-methyl-D-aspartate receptor encephalitis (anti-NMDAR encephalitis) is a newly identified autoimmune encephalitis associated with antibodies against functional NMDA receptors that predominantly affects young females and exhibits a well defined set of clinical features [1]. Unlike classic paraneoplastic limbic encephalpathies with onconeural antibodies directed to intracellular antigens, anti-NMDAR encephalitis harbors antibodies against neuronal extracellular membrane N-methyl-D-aspartate receptor subunit 1 (NR1) of NMDA receptor, and may not be accompanied with tumors [2]. It has been demonstrated that anti-NR1 antibodies selectively bind, cross-link, and internalize surface NMDA receptors, and lead to decreased postsynaptic NMDA receptor-mediated currents in a reversible and antibody titer-dependent manner [3]. Although recent studies showed few patients with non-tumor-associated anti-NMDAR encephalitis have evidence of elevated anti-thyroid peroxidase (anti-TPO) antibodies [4-7], there is lack of anti-NMDAR and anti-TPO antibodies combined follow-up in details in current literatures. The majority of patients with anti-NMDAR encephalitis have a prodromal flu-like illness. In line with this, numerous pathogens have been implicated and identified on serum studies, including mycoplasma pneumoniae [4,5], influenza virus A, influenza virus B, Chlamydia pneumoniae, Bordetella pertussis and parapertussis [7]. To our knowledge, this represents the first anti-NMDAR encephalitis case associated with serum Epstein-Barr virus viral capsid antigen IgM (EBV-VCA-IgM).

## Case presentation

An otherwise healthy 17-year-old urban high school girl was brought to the emergency room for episodes of generalized tonic-clonic convulsions of all extremities. She was described to have auditory hallucination by complaining about the "noisy" electric wire in her bedroom three days ago. She had no history of tobacco, alcohol, or drug use. The family history and previous illnesses of the patient were unrevealing. Upon arrival, her axillary temperature was 37.6°C. She was not oriented to person, place or time. She couldn't recall what teachers had taught few hours ago and had difficulty in performing serial 7's. Standard blood screening tests revealed increased white blood cell count (15.66 × 10^9^/L, normal range 4~10 × 10^9^/L) but normal lymphocyte count (1.44 × 10^9^/L, normal range 0.8~4 × 10^9^/L). Cerebrospinal fluid (CSF) analyses were unremarkable, including antibodies panel of anti-thyroglobulin (TG) antibodies (4.6 U/ml, normal range 0~60 U/ml, radioimmunoassay), anti-TPO antibodies (18.1 U/ml, normal range 0~60 U/ml, radioimmunoassay) and negative EBV-VCA-IgM (enzyme-linked immunosorbent assay, ELISA). Blood and urine screenings for drug abuse and toxication were negative. She was admitted to the neurology ward; acyclovir (1500 mg IV QD) was started for empiric treatment of viral encephalitis.

**Figure 1:**
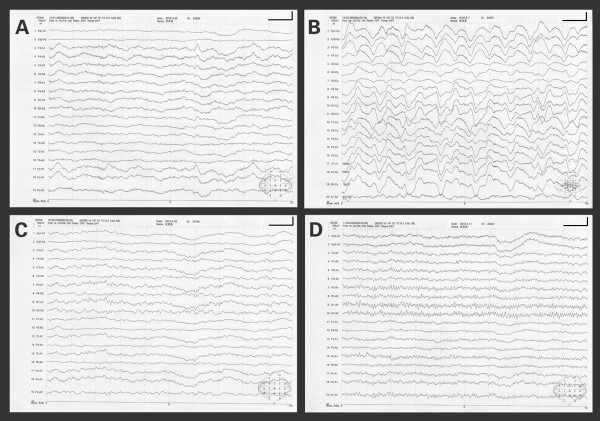
**Serial electroencephalographies of the patient without sedation**. (A) Recording on day 2 showed background rhythm was taken place by abnormal diffuse low-voltage fast-activities, when patient was alert but with psychiatric symptoms. (B) Recording on day 11 revealed aberrant universal delta activity, when patient was in coma. (C) Recording on day 34 (8^th ^day after teratoma removal) demonstrated the return of background alpha rhythm with few theta activity, when the patient's conscious was recovered without oral-facial dyskinesias or extremities convulsions. (D) Recording on day 44 (18^th ^day after teratoma removal) showed a normal alpha activity. The calibration marks represent 1 second and 100 μV.

After further investigation, her EBV-VCA-IgM, EBV-VCA-IgG and EBV nuclear antigen IgG (EBNA-IgG) seropositivities were identified with ELISA. Serum tumor markers, antinuclear antibodies, anti-extractable nuclear antigen antibodies and anti-Hu, Yo, Ri antibodies were all negative or within normal limits. Magnetic resonance imaging (MRI) of brain was normal. Electroencephalography (EEG) on day 2 demonstrated abnormal diffuse low-voltage fast-activities (Figure 1A), when patient was alert but with psychiatric symptoms, saying"How can I become taller? My father is short of money ! I'm going to die !" Radioimmunoassay showed markedly elevated anti-TG (138 U/ml, normal range 0~60 U/ml) and anti-TPO (> 1300 U/ml, normal range 0~60 U/ml) antibodies in her serum, yet her serum T3, FT3, T4, FT4, TSH levels along with thyroid ultrasonography were normal. Over the next few days, the patient's consciousness level gradually decreased. She then developed occasional lip licking and tongue protrusion with convulsions of all extremities. Valproic acid and oxcarbazepine were used successively and then simultaneously, however, her oral-facial dyskinesias and extremities convulsions persisted despite the use of dual anticonvulsant therapy. Repeat lumbar puncture on day 6 revealed elevated CSF opening pressure (21 cm H_2_O, normal range 8~18 cm H_2_O), protein concentration (58.0 mg/dl, normal range 20~40 mg/dl) and pleocytosis (73 × 10^6^/L, normal range 0~10 × 10^6^/L) as well.

**Figure 2:**
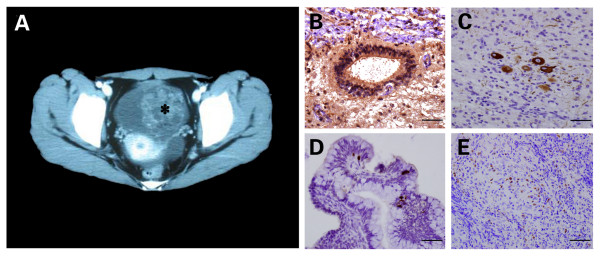
**Computed tomography of the pelvis (A) revealed a cystic left adnexal mass with an internal focus of fat and high attenuation material suggesting an ovarian teratoma (asterisk)**. N-methyl-D-aspartate receptor subunit 1 (NR1) immunostain demonstrated primitive neural tubes (B) within teratoma, while neurofilament immunostain showed mature ganglion cells (C). Epstein-Barr virus encoded RNA (EBER) *in situ *hybridization revealed scattered latent EBV infected cells in mucinous glandular epithelium of the teratoma (D, brown color), an EBV infected NK/T cell lymphoma (E) served as positive control. (For panels B to E, bars = 50 μm).

Routine abdominal and pelvic ultrasound on day 5 revealed a cyst in the left adnexa, which was 10 cm in diameter. The pelvic computed tomography scan with enhancement next day showed a calcified hypodense cystic lesion (10.5 cm × 6.2 cm × 6.0 cm) and an ovarian teratoma was suspected (Figure 2A). From day 7 on, the patient received intravenous immunoglobulin (0.4 g/kg body weight/day, 5 days), followed by intravenous methylprednisolone (500 mg/day, 3 days). Another EEG on day 11 demonstrated an aberrant universal delta activity (Figure 1B) when the patient was in coma. On day 17, the patient presented hypersalivation and sinus tachycardia (ranging from 100~130 beats/min) without hyperthermia and convulsions. On day 19, her arterial blood gas analysis suggested CO_2 _retention. She was intubated and mechanically ventilated. On day 21, she could follow simple commands such as blinking eyes and grasping fists. From day 22 on, her consciousness started to recover with less frequent paroxysmal events (ie. oral-facial dyskinesias and extremities convulsions) happened. A left salpingooophorectomy was performed on day 26 and the mass was completely removed. The pelvic lymph nodes and peritoneal washings were negative. Final pathology diagnosis was immature cystic teratoma of the left ovary, stage I, and grade 1.

One day following resection of the teratoma, the patient was successfully weaned from the ventilator. Paroxysmal events had also been controlled with valproic acid and oxcarbazepine. On day 32 (the 6^th ^day after operation), her consciousness was fully recovered, short-term memory, orientation and calculation examinations were also normal. On day 34 (the 8^th ^day after operation), patient's EEG demonstrated the return of background alpha rhythm with few theta activity (Figure 1C) and she could speak fluently without lip licking and chewing. At the same time, antibodies to NR1 were identified in patient's CSF.

Fifty-seven days following onset of symptoms and 54 days following admission, the patient was discharged, symptom free. Repeat MRI and EEG were normal (Figure 1D). Serum anti-TG antibodies (361 U/ml, normal range 0~60 U/ml) and anti-TPO antibodies (> 1300 U/ml, normal range 0~60 U/ml) remained elevated.

### Pathological findings of immature ovarian teratoma

Both NR1 positive primitive neural tubes (Figure 2B) and neurofilament positive ganglion cells were found in the teratoma (Figure 2C).

*In situ *hybridization (ISH) for detecting latent EBV infection in teratoma was performed with the Epstein-Barr virus encoded RNA (EBER)-probe ISH kit (Triplex, Fuzhou, China) according to the manufacturer's instructions. Dark brown nuclear staining identified a positive hybridization. In current case, EBER ISH revealed scattered latent EBV infected cells in mucinous glandular epithelium of the teratoma (Figure 2D), an EBV infected NK/T cell lymphoma (Figure 2E) served as positive control.

### One year follow-up of patient's antibodies in serum

During one year follow-up of the patient, her physical examinations and EEG were unremarkable. Her serum was sent for conventional tests for anti-TPO, anti-TG antibodies with radioimmunoassay and EBV-VCA-IgM with ELISA. Meanwhile, patient's serum IgG antibodies against TPO, TG, NMDAR, EBV-VCA, EBV early antigen (EBV-EA), EBNA, IgG avidity of EBV-VCA and IgM antibodies against EBV-VCA were also detected by indirect immunofluorescence assays according to the manufacturer's recommendations (Euroimmune AG, Lübeck, Germany). Another Hashimoto thyroiditis patient's serum with high titers of both anti-TPO and anti-TG antibodies confirmed by radioimmunoassay was employed as positive control in indirect immunofluorescence assay.

Radioimmunoassay showed normal anti-TG antibodies (51.8 U/ml, normal range 0~60 U/ml). Anti-TPO antibody level remained elevated (> 1300 U/ml, normal range 0~60 U/ml). Serum EBV-VCA-IgM was negative by ELISA.

**Figure 3:**
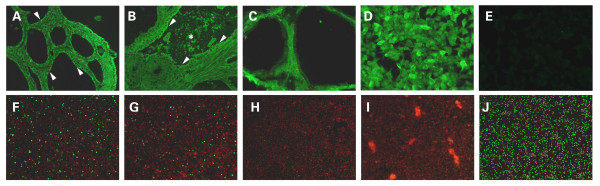
**Indirect immunofluorescene assays with patient's serum obtained one year after symptoms onset (dilution 1:10) revealed anti-TPO antibodies specific granular staining in the cytoplasm of the follicle epithelium (A, white arrowheads)**. Serum of another Hashimoto thyroiditis patient served as positive control showed both granular staining in the cytoplasm of the follicle epithelium (B, white arrowheads) and coarse staining in the colloid of follicles (B, white asterisk), which represented anti-TPO and anti-TG seropositivity, respectively. Phosphate buffered saline solution added as negative control gave neither of the above mentioned fluorescence pattern (C). Positive staining was observed after human embryonic kidney cells transfected with NMDA receptors was incubated with patient's serum (dilution 1:10), confirming the continuing presence of autoantibodies against NMDA receptors (D), while patient's serum didn't react with untransfected human embryonic kidney cells (E). As for the EBV associated serostatus, EBV-VCA-IgG (F), high avidity EBV-VCA-IgG (G) and EBNA-IgG (J) were present in patient's serum, while EBV-VCA-IgM (H) and EBV-EA-IgG (I) were absent, suggesting a chronic EBV infection without relapse or reactivation during follow-up. (Magnifications: 10 × F~J, 20 × A~C and 40 × D~E).

In indirect immunofluorescence assays, the patient's serum autoantibodies against TPO made a granular staining in the cytoplasm of the follicle epithelium (Figure 3A, white arrowheads). Serum of a Hashimoto thyroiditis patient served as positive control, showing both granular staining in the cytoplasm of the follicle epithelium (Figure 3B, white arrowheads) and coarse staining in the colloid of follicles (Figure 3B, white asterisk), which represented anti-TPO and anti-TG seropositivity, respectively. Phosphate buffered saline (PBS) solution added as negative control gave neither of the above mentioned fluorescence pattern (Figure 3C). Positive staining was observed after human embryonic kidney cells transfected with NMDA receptors was incubated with patient's serum, confirming the continuing presence of autoantibodies against NMDA receptors (Figure 3D), while patient's serum didn't react with untransfected human embryonic kidney cells (Figure 3E). As for the EBV associated serostatus, EBV-VCA-IgG (Figure 3F), high avidity EBV-VCA-IgG (Figure 3G) and EBNA-IgG (Figure 3J) were present in patient's serum, while EBV-VCA-IgM (Figure 3H) and EBV-EA-IgG (Figure 3I) were absent, suggesting a chronic EBV infection without relapse or reactivation.

## Discussions

Our case followed a classic clinical picture of anti-NMDAR encephalitis resulting from the loss of NR1 subunit of NMDA receptors as presented in the current literatures, including psychotic behavior, disruption of dopaminergic pathway (orofacial movement), dysautonomia (cardiac dysrhythmia, hypersalivation, central hypoventilation) and memory deficits are similar to those obtained with models of genetic or pharmacologic attenuation of NMDA receptors function [8-10]. High titers of serum anti-TPO antibodies suggested comorbid diagnosis of Hashimoto encephalopathy (HE) requires further attentions. The presence of serum EBV-VCA-IgM (not previously described in patients with anti-NMDAR encephalitis) provides further evidence that anti-NMDAR encephalitis is not merely a paraneoplastic disorder, but that a probable postinfectious autoimmue process contributes to disease presentation.

HE is a steroid-responsive encephalopathy associated with elevated blood concentrations of anti-thyroid antibodies and euthyroid or mildly hypothyroid function. Alink summarized 25 pediatric HE cases mainly with acute manifestations, among which the most frequent symptoms were seizures (80%), confusion (52%), hallucinations (32%), and only half of the patients develop abnormal neuroimaging [11]. The diagnosis of HE requires both detection of high concentrations of anti-TPO antibodies in serum, and exclusion of other toxic-metabolic encephalopathies. From above mentioned aspects, the current case met the diagnostic criteria for HE [12] until both the ovary immature teratoma and anti-NMDAR antibodies were identified. In the present case, serum anti-TPO antibodies remained elevated one year after discharge, despite resolution of clinical symptoms, further suggesting that the likely diagnosis in this case was anti-NMDAR encephalitis. Although, anti-TPO antibodies directed at microsomes and released from damaged thyroid cells were found in all HE cases, they have also been found in autoimmmue diseases ranging from type 1 diabetes mellitus to rheumatoid arthritis [13,14], even in euthyroid individuals [15]. Hence, clinicians should be cautioned against accepting HE as the sole explanation for an encephalitic illness, after excluding conventional metabolic, infectious or vascular etiologies, and merely based on anti-TPO test. On the other hand, with evolving case studies of anti-NMDAR encephalitis, more and more authors regard HE as an important differential diagnosis [16-20]. Recently, Tüzün and colleagues compared serum autoantibodies to neuronal surface antigens in serum thyroid antibody-positive and -negative limbic encephalitis, suggesting patients with anti-thyroid antibodies are inclined to develop anti-neuronal immune responses and autoimmune encephalitis such as anti-NMDAR encephalitis, which supported the notion that neuronal and thyroid autoimmunities might represent a pathogenic spectrum [21]. Interestingly, although prednisone is known to reduce thyroid antibodies titers, several courses of immunotherapy had no effect on sera anti-TPO antibodies reduction in current case. Her serum anti-TPO antibodies were still elevated when she was discharged and had persisted for a year after dismissal, which suggested a propensity for autoimmunity in anti-NMDAR encephalitis.

A high incidence of prodromal hyperthermia and viral-like symptoms during the course of anti-NMDAR encephalitis frequently lead to extensive studies to rule out infections [2,22]. Although some positive infectious serology has been reported in anti-NMDAR encephalitis cases, not a common pathogen has been identified even in cases without tumors [23]. The preceding active EBV infection or reactivation suggested by EBV-VCA-IgM in this case hasn't been previously reported.

In developing countries like China, primary EBV infection is usually asymptomatic and occurs through close contacts between parents and children within the first 3 years of life [24]. Therefore, given lack of typical EBV infection symptoms and blood lymphoctosis, EBV-VCA-IgM seropositivity of current adolescent case on admission was not due to primary EBV infection but probably recent EBV reactivation. In addition, evidence of acute hepatitis A virus or HIV infections, which had been reported to have false positive reactions of the EBV-VCA-IgM ELISA was absent in this patient [25]. Follow-up EBV-VCA-IgM seroreversion proved by both ELISA and indirect immunofluorescence assay which is known as gold standard for highly sensitive and monospecific antibody detection [26], further excluded the persisting EBV-VCA-IgM seropostive status and reinforced the credibility of our conventional ELISA methodology. It is well acknowledged that the titer of CSF anti-NMDAR antibodies appears to correlate more closely with the clinical outcome than serum antibodies and prolonged follow-up studies have found anti-NMDAR antibodies in CSF and serum [2,3,27,28]. Depriving NR1 as autoantigen with teratoma and reducing anti-NMDAR antibody titers by previous immunotherapies may explain the prolonged symptom-free intervals in those cases. Taking into account different EBV-VCA-IgM serostatus from admission to one year follow-up, we proposed the prodromal EBV reactivation and following cascade may also contribute to triggering and boosting the immune response or facilitate autoantibodies crossing the blood-brain barrier in our patient one year ago [23]. Controlled studies are expected to compare the concomitant infectious or inflammatory status of patients with and without prodromal flu-like symptoms and may shed new light on the pathophysiology of anti-NMDAR encephalitis.

In chronic aspects, persistence and reactivation of EBV is presumed to elicit autoimmunity in multiple sclerosis, systemic lupus erythematosus, rheumatoid arthritis, myasthenia gravis, Hashimoto thyroiditis, etc [24,29,30], but hasn't been linked with anti-NMDAR encephalitis up to now. Following primary infection, EBV latency is maintained by expression of a set of viral proteins that deliver activation, growth, and survival signals to infected B cells. These B cells are able to cross the blood-brain barrier, and are believed to undergo restimulation, antigen-driven affinity maturation, clonal expansion, and differentiation into antibody-secreting plasma cells which may cause damage in self-tissues [31]. EBV has also been associated with numerous cancers such as Burkitt's lymphoma, Hodgkin lymphoma, nasopharyngeal carcinoma, gastric adenocarcinoma and even breast cancer [24], the latent EBV infection in this immature teratoma proved by EBER ISH may either suggest oncogenicity of EBV in teratoma or underlying EBV related immunopathogenesis of anti-NMDAR encephalitis as we proposed previously.

The improvement in the clinical presentation with therapies also can be correlated to the resolution of the EEG changes (Figure 1A-D). Although the patient was lack of severe stressors and functioned very well in daily life before falling ill, she presented clinical spectrum of seizures, agitation, autonomic instability and hypoventilation compatible with a catatonic disorder [32,33]. Nevertheless, without aggressive management of the observable symptoms of catatonia, the most profound improvements in psychiatric and behavioral symptoms occurred after the teratoma had been resected. Unlike recently reported two non-tumor associated anti-NMDAR encephalitis cases with anti-TPO and infectious serology concurrence [7], the patient had complete clinical recovery both on discharge and one year later, which may also due to timely tumor resection. However, controlled studies and more cases are still necessary to prove the real effectiveness of IV immunoglobulin and IV steroids recommended as first line immunotherapies by some authors [19,26] in anti-NMDAR encephalitis. Factors related to the surgery including anesthetics or induction of sleep may not be account for patient's clinical improvements, for propofol and midazolam administered during endotracheal intubation hadn't change the course of her illness.

## Limitations

Due to the limited amount of teratoma tissue available, we could not further characterize whether tumoral cells or infiltrating lymphocytes were positively EBER hybridized. Thus, from the data provided we couldn't distinguish local (specific and neoplastic) versus systemic (nonspecific and lymphocytic) EBV latent infection in the teratoma. On follow-up assessment, our patient refused repeat lumbar puncture limited our ability to comment on CSF anti-NMDAR antibody titer.

## Conclusions

In summary, persistent high titers of serum anti-thyroid peroxidase antibodies from admission to discharge and until one year later in our patient may not only suggest a propensity to autoimmunity in anti-NMDAR encephalitis, but support the notion that neuronal and thyroid autoimmunities represent a pathogenic spectrum. The enduring anti-NMDAR antibodies positivity from admission to one year follow-up, EBV latent infected ovarian teratoma, and seroreversion of EBV-VCA-IgM of current case might also raise the issue of elucidating the triggers and boosters of this autoimmune encephalopathy in the future.

## Consent

Written informed consent was obtained from the patient for publication of this case report and accompanying images. A copy of the written consent is available for review by the Editor-in-Chief of this journal.

## List of abbreviations

anti-NMDAR encephalitis: anti-N-methyl-D-aspartate receptor encephalitis; NR1: N-Methyl-D-aspartate receptor subunit 1; EBV-VCA-IgM: Epstein-Barr virus viral capsid antigen IgM; anti-TPO antibodies: anti-thyroid peroxidase antibodies; anti-TG antibodies: anti-thyroglobulin antibodies; ELISA: enzyme-linked immunosorbent assay; HE: Hashimoto encephalopathy; EBV: Epstein-Barr virus; CSF: Cerebrospinal fluid; MRI: magnetic resonance imaging; EEG: electroencephalography; EBER: Epstein-Barr virus encoded RNA; ISH: *in situ *hybridization; EBV-EA: EBV early antigen; EBNA: EBV nuclear antigen.

## Competing interests

The authors declare that they have no competing interests.

## Authors' contributions

CLX, LL, WQZ, JWW participated in collecting clinical data, EEGs and pathological findings as well as drafting the manuscript. All authors contributed in the evaluation and care of the patient and revising of the manuscript. All authors read and approved the final manuscript.

## Authors' information

All authors are clinical neurologists. LL and WQZ are also the members of Division of Neuropathology, Department of Neurology, Beijing Friendship Hospital, Capital Medical University headed by WQZ. JWW is the head of Division of Neuroimmunology and Neurological Infections, Department of Neurology, Beijing Friendship Hospital, Capital Medical University. JML is the director of the Department of Neurology, Beijing Friendship Hospital, Capital Medical University.

## Pre-publication history

The pre-publication history for this paper can be accessed here:

http://www.biomedcentral.com/1471-2377/11/149/prepub
